# A conceptual model of treatment burden and patient capacity in stroke

**DOI:** 10.1186/s12875-017-0691-4

**Published:** 2018-01-09

**Authors:** Katie I. Gallacher, Carl R. May, Peter Langhorne, Frances S. Mair

**Affiliations:** 1 Institute of Health and Wellbeing, University of Glasgow, 1 Horselethill Road, Glasgow, G12 9LX Scotland; 2 Health Sciences, University of Southampton, Southampton, England; 3 Institute of Cardiovascular and Medical Sciences, University of Glasgow, Glasgow, Scotland

**Keywords:** Stroke, Treatment burden, Patient capacity, Patient experience, Qualitative

## Abstract

**Background:**

Treatment burden is the workload of healthcare experienced by those with long-term conditions and the impact that this has on well-being. Treatment burden can negatively impact on quality of life and adherence to treatments. Individuals are likely to differ in their ability to manage health problems and follow treatments, defined as patient capacity. This has been under investigated in stroke. The aim of this paper is to create a conceptual model of treatment burden and patient capacity for people who have had a stroke through exploration of their experiences of healthcare.

**Methods:**

Interviews were conducted at home with 29 individuals who have had a stroke. These were recorded and transcribed verbatim. Fifteen explored treatment burden and were analysed by framework analysis underpinned by Normalisation Process Theory (NPT). Fourteen explored patient capacity and were analysed by thematic analysis. Taxonomies of treatment burden and patient capacity were created and a conceptual model produced.

**Results:**

Mean age was 68 years. Sixteen were men and 13 women. The following broad areas of treatment burden were identified: making sense of stroke management and planning care; interacting with others including health professionals, family and other stroke patients; enacting management strategies; and reflecting on management. Treatment burdens were identified as arising from either: the workload of healthcare; or the endurance of care deficiencies. Six factors were identified that influence patient capacity: personal attributes and skills; physical and cognitive abilities; support network; financial status; life workload, and environment.

**Conclusions:**

Healthcare workload and the presence of care deficiencies can influence and be influenced by patient capacity. The quality and configuration of health and social care services has considerable influence on treatment burden and patient capacity. Findings have important implications for the design of clinical guidelines and healthcare delivery, highlighting issues such as the importance of good care co-ordination.

**Electronic supplementary material:**

The online version of this article (10.1186/s12875-017-0691-4) contains supplementary material, which is available to authorized users.

## Background

Treatment burden is the workload of healthcare for patients and the effects of this on well-being [1, 2]. Excessive treatment burden can lead to negative outcomes such as reduced quality-of-life, non-adherence and wasted resources [3, 4]. Patients vary in their abilities to follow treatments and engage with health professionals depending on a variety of physical, psychological and social factors, defined as patient capacity [5, 6]. The configuration of health services and recommendations in clinical guidelines may exacerbate treatment burden. This is a significant problem for patients that could be amenable to change through proper exploration and action by those responsible for healthcare provision [3, 4]. There has been growing interest in treatment burden in those with chronic disease [2, 7–10] however there has been limited exploration in people with stroke [11, 12]. Two important conceptual models of treatment burden in those with chronic illness have been created: Burden of Treatment Theory (BoTT) [6] and the Cumulative Complexity Model (CuCoM) [5]. Both of these models highlight the influences of healthcare workload, patient capacity and the provision of health services on treatment burden. Exploration of treatment burden and patient capacity in a stroke population is important as there are likely to be stroke-specific difficulties that remain undetected in studies of other conditions e.g. aphasia related difficulties.

We have previously created a taxonomy of treatment burden in stroke through systematic review of the qualitative literature [11]. This review reported a lack of primary studies that aimed to explore treatment burden in stroke and described four aspects of treatment burden: making sense of stroke management and planning care; interacting with others; enacting management strategies; and reflecting on management. The aims of this paper are: 1) to expand and verify our taxonomy of treatment burden through interviews with people who have had a stroke; 2) to explore the factors that influence capacity in those affected by stroke; and 3) to create a conceptual model of treatment burden and patient capacity in stroke.

## Methods

Ethical approval was granted by the West of Scotland Research Ethics Service (11/AL/0266).

### Recruitment of participants

Twenty-nine participants who had previously had a stroke were recruited from a single health board area in Scotland. Eighteen were recruited from primary care and 11 from secondary care. Participants were either sent a letter in the post or a research nurse handed them an information pack at the stroke clinics or stroke wards at three hospitals in Glasgow (The Western Infirmary, Glasgow Royal Infirmary and Stobhill Hospital). Those recruited in primary care were identified through a search of primary care practice registers for a diagnosis of stroke.

Recruitment was purposive to include a range of patient characteristics including gender, age, deprivation, and severity of disability. Recruitment was conducted in both primary and secondary care in order to include individuals undergoing acute management and others receiving longer-term care. Those who contacted the research team were screened for desirable characteristics and to ensure they met inclusion and exclusion criteria (shown in Table 1). For example, if there was a predominance of elderly participants then younger ones were sought. Participants with aphasia were included, during the interviews alternative methods of communication were used as necessary and carers often spoke with non-verbal verification from the patient.

**Table 1:** Inclusion and exclusion criteria for participants

Inclusion	Exclusion
18 years and over	A history of mental impairment that would suggest that they would be unable to give informed consent to participate in the study
Diagnosis of haemorrhagic or ischaemic stroke	Unable to communicate in English
	A history of violence towards members of the primary health care team or other health professionals
	A terminal illness, other than stroke, with life expectancy less than 6 months

### Data collection

Semi-structured interviews lasting approximately 1 h were undertaken in participant homes. In the first 15 interviews, participants were asked to describe the care they had received for their stroke and any burdens they had encountered. Data analyses suggested saturation had been reached; therefore, during the next 14 interviews, burdens already identified were presented to the patient for verification and comment, and any new burdens sought. Additionally, participants were asked to explain factors that had increased or decreased their capacity to cope with their treatments. Interview schedules are provided in Additional files 1 and 2. For exploration of capacity, it was felt that data saturation had been reached after 14 interviews therefore no more were conducted. Many participants opted to have carers present during the interviews, in these cases the focus remained on the effect of treatments on the person with stroke and data on carer burdens were not analysed. Field notes were taken during the interviews. Interviews were digitally recorded, with participant consent, and then transcribed verbatim.

For all participants an assessment of disability was made using the Modified Rankin Scale [13] and socioeconomic status was measured using the Scottish Index of Multiple Deprivation (SIMD) calculated from the participant’s postcode using an online tool (http://www.sns.gov.uk/). Other data gathered from primary care records included: time since last stroke; number of strokes; number of TIAs; number of comorbidities; and number of regular medications (issued in the past 3 months).

### Data analysis

#### First fifteen interviews

Data from the first 15 interviews were analysed using a coding framework informed by Normalisation Process Theory (NPT) [14]. NPT is a sociological theory that seeks to explain how a set of practices such as those involved in stroke management are implemented, embedded and integrated into everyday life [15, 16] and has been used previously to examine the issue of treatment burden [1, 11]. NPT is built on four constructs that organize the patient workload of chronic disease management into the following broad categories: sense-making; interacting with others; enacting management strategies; and appraisal work.

Data analysis was facilitated by Nvivo 10 software. The five stages of framework analysis were followed: familiarisation, identifying a thematic framework, indexing, charting, mapping and interpretation [17–20]. During analysis the coding framework was scrutinised for any necessary modifications, but none were necessary. A careful note was made of any treatment burdens that fell outside the coding frame, in order to assess if the framework was ‘fit for purpose’.

The first author coded all transcripts. To enhance reliability of coding, four transcripts were also coded independently by another author, and any differences discussed. No major conflicts arose.

### Second fourteen interviews

Thematic analysis was used to code data from the second 14 interviews. This was conducted to allow confirmation of data saturation from the framework analysis, and additionally to explore factors that influence patient capacity (after discussion it was decided that the NPT based framework would not have been suitable for this purpose). During data analysis the six stages of thematic analysis were followed: familiarization with data, generating initial codes, searching for themes among codes, reviewing themes, defining and naming themes, and producing the final report [21].

#### Overall analysis and creation of the conceptual model

Themes from all interviews were sorted into those that described treatment burden and those that described factors that influence capacity. Components of treatment burden found in the second 14 interviews were carefully compared to and merged with findings from the first 15 i.e. themes with similar meaning were combined and new or contradictory ones sought. The results from all 29 interviews were then similarly compared to findings from the previous systematic review [11], and a taxonomy of treatment burden in stroke created. A separate taxonomy of patient capacity was created. The two taxonomies were then examined for causal pathways and a conceptual model constructed.

## Results

### Participants

Mean age of participants was 68. Sixteen were male and 13 female. Participants were registered at 18 different primary care practices in one health board area in Scotland. Additional file 3 provides participant details.

### Treatment burden

Treatment burdens were identified as arising from either: 1) the workload of healthcare; or 2) the endurance of care deficiencies. Healthcare workload encompassed the acts of thinking, organising, doing and reflecting that occur during the management of stroke. Care deficiencies were aspects of health or social care that did not meet perceived needs or expectations. The taxonomy of treatment burden is shown in Table 2 under four phases of stroke management that reflect the NPT domains: making sense of stroke and planning care; interacting with others; enacting management strategies; reflecting on management. No data fell outside these categories.

**Table 2:** Taxonomy of treatment burden in stroke

Type of treatment burden	Healthcare workload	Care deficiencies
Making sense of stroke management and planning care	• Understanding symptoms, investigations, treatments, risk factors • Information gathering • Taking responsibility • Goal setting & prioritising • Problem solving • Managing uncertainty & maintaining motivation • Developing coping strategies • Coping with negative emotions	• Lack of information provision & poor signposting • Information hard to understand • Poorly timed information • Not enough verbal information • Not tailored to individual • Lack of support with care planning
Interacting with others	• Seeking advice or help from health and social care professionals • Gaining support from friends, family, fellow patients • Strained relationships • Protecting carers • Stigma	• Misdiagnosis • Paternalism • Lack of understanding • Mismatch in ideas • Poor access to GP • Poorly co-ordinated care • Poor continuity • Poor communication from GP
Enacting management strategies	• Acute care • Inpatient rehabilitation • Discharge home or to care home • Community rehabilitation • Outpatient appointments • Medications • Risk factor modification • Co-morbidities • Adaptations to home • Home care • Return to driving and employment • Mobility aids • Finances • Enacting coping strategies • Psychological adjustment • Alternative therapies	• Waiting times as inpatient • Unpleasant ward • Poorly supported discharge • Poor GP follow up • Poor follow up for milder cases • Lack of help with transport to appointments • Complicated medication regimes • Poor access to home adaptations and walking aids • Substandard home care • Poor access to driving assessment • Complicated benefits system • Lack of psychological support and support groups
Reflecting on management	• Routine appointments for review • Joint healthcare decisions • Reflecting on progress • Non-adherence • Keeping up to date • Worry about another stroke	• Lack of review for milder cases • Poor long term follow up for all

Treatment burden findings are described below with exemplar quotes.

#### Making sense of stroke and planning care

##### Healthcare workload

Participants described the challenge of making sense of stroke symptoms before they sought medical help. Post diagnosis they were given a mixture of written and verbal information.

*See I was under the impression before this happened to me that a stroke meant something to do with the heart or something like that. I didn’t know exactly what it was. And when I started to get information I realised what had happened to me medically…I’d say for the first day, twenty four hours it was hard to take it all in. (Participant 9)*

Participants had to work to make sense of different types of stroke, investigations, treatments, and the roles of different health professionals. They had performed self-directed research to collect information.

*We did (look up information) because with the stroke thing we were up there, they send you quite a bit of information. The one that we are affiliated to…chest, heart and stroke. I’ll show the book I've got one in there, the magazine things. (Participant 7)*

Many described spending time planning their recovery, which included setting and prioritising goals. Participants described cognitive processing such as problem solving, dealing with uncertainties of how well they would recover, and maintaining motivation.

*I think it was will power with me that brought me back to life. Will power I think it was to get me back you know so I got up and I done it even though I didn’t feel you know I still done it you know. (Participant 16)*

Enduring care deficiencies

Information provision at the time of stroke was variable, but most felt they received inadequate information about investigations, treatments, risk factor modification, follow up, services available to them on discharge, and signs of another stroke.

*No I don’t think they spoke enough. They never let you know what was, exactly what was happening. And you didn’t get much feedback off them… they just don’t give you any information on how its, how to avoid it, how it’s happening, stress things like that. They just don’t tell you anything. It’s just a case of take these tablets. And you’ll be all right. (Participant 13)*

Some felt the information they were given was difficult to understand and poorly timed. Many found written information helpful, but accompanied by insufficient verbal information from health professionals. Some participants appeared confused about where to find information themselves after discharge from hospital. Interestingly, several participants, mostly female, preferred to not be given information about their stroke in case this caused anxiety.

*I don’t, I don’t look into anything because I think in my mind what I don’t know I can’t think about. (Interview 22)*

Participants less severely affected by their stroke tended to feel the least supported by health services with regards care planning.

*Okay I was a very mild case; I’m not complaining bitterly that nobody was there to back me up. But I feel it would have been good for someone to say hey we’ve got a couple of wee tests here just, let’s see how you do this. And say yeah you are doing it better than you did three months ago or two months ago and there was nothing. (Participant 15)*

#### Interacting with others

##### Healthcare workload

On the hospital ward, participants interacted with a range of medical and nursing staff and therapists. In the community, some were referred onto the community stroke team, which included a range of therapists who continued treatment for a fixed time following discharge. The primary care doctor and nurse were the main points of contact thereafter. Most participants reported contacting their family doctor regularly for advice. The majority contacted the clinic by telephone and then either took advice over the phone, organised a clinic appointment or made arrangements for a home visit. Several patients had contacted out-of-hours primary care services at some point.

*See I don’t phone the surgery I go down there for half past eight. Because if I phoned looking for an appointment for that day, I've no car now so if she said I’ll give you an appointment for quarter to nine I would never make it down for quarter to nine so I go down there and wait for them opening and… I get an early appointment. (Participant 6)*

Some reported negative interactions with family members due to the strain of managing their stroke. Participants did not always like to feel reliant on others, and many did not like the stigma attached to needing care.

##### Enduring care deficiencies

Some participants reported misdiagnosis at initial presentation of stroke. Some had sought help from a medical professional on more than one occasion before stroke was diagnosed, with unsatisfactory outcomes.

During the hospital stay, nursing treatment on the acute stroke ward was described by most as excellent. However, experiences varied between hospitals and also between wards, with some describing care that did not meet their expectations. One participant had been offered lunch before she had been given a swallowing assessment leaving her at risk of choking on her food, and another complained that one of the nurses had made several mistakes when checking blood pressure and administering medications.

*And then in the morning when she came round to give us the …say for instance I get six there was only four and I said to her there is only four there I think I should get six and she went oh well what one’s is it that’s not there, that’s missing and I went I don’t know… and I had to wait for the staff nurse to come in. (Participant 20)*

Several participants reported a lack of time with clinical staff during their hospital admission, leaving them feeling isolated on the ward, particularly at weekends. Despite this, most spoke highly of their therapists on the ward and in the community, with very rare reports of unsatisfactory encounters.

Many participants spoke highly of their family practitioner, but some reported unsatisfactory interactions, usually in the form of receiving treatment or advice they felt was inappropriate, lacking in empathy, or too paternalistic. One participant complained about poor communication from her family doctor, in that she had not been informed about a change in her medications.

*But they advised me to come off the amiodarone but that was all they said, they said they would write to my doctor. So the next thing I knew there was two items on my prescriptions. But I had, I had never heard of them you see and I thought I don’t think these are mine you know. (Participant 6)*

Several participants reported incidents involving poor communication between their family doctor and other health or social care professionals. One man was nearly prescribed harmful medication due to poor information exchange between his family practitioner and pharmacist.

*There was a chemist I had to go down and see at the health centre one day and he wanted to discuss my medication. And he said you are needing to be on, they took me off aspirin when I had the stroke and he said you need to be on aspirin, I said no I think from what they said that caused me the problem, oh no you definitely need to be on aspirin and I said well I’m not going to take any I said you better go and check up on that so he went and I don’t know who he spoke to and he came back and he said you are right you shouldn’t be on aspirin because I had a bleed. (Participant 2)*

Most described their primary care appointment system as satisfactory but some described waiting times of 2 weeks or more.

Of those who had requested their family doctor to visit them at home, most did not report difficulties. One man had not had an annual review of his medications in several years as he could not attend the surgery and had struggled to arrange a home visit for this.

*If you can get him to come out to the house you know, a lot of them don’t come to the house you know. Yes to get there you know. (Participant 12)*

Not all patients felt that seeing the same doctor was important, but some described continuity in primary care as lacking. Access to the out of hours health care service were also described as difficult by some. This was generally considered worse than access to services during working hours.

With regard to hospital outpatient appointments, a few mentioned that they had often seen doctors in training rather than the consultant, meaning that continuity had been lacking. Some participants described receiving conflicting information from different health professionals.

*Yeah now the chemist said that it was all right to take the statin in the morning as well, my other doctor said he thought it was better at night. (Participant 20)*

### Enacting management strategies

#### Institutional admissions

##### Healthcare workload

Participants reported hospital admissions of varying lengths of time, ranging from several days to months. Most had received initial care on an acute ward, followed by transfer to a rehabilitation ward for subsequent therapies, often in another hospital. One man was transferred to a different city during the acute phase of treatment as his family had moved house during his hospital stay.

Soon after admission, patients had generally undergone assessment by a physiotherapist, occupational therapist, speech and language therapist, and dietician. Patients described working hard to achieve goals.

*But I made my mind up that I was going to get up and walk again and I just pushed myself and pushed myself. (Participant 2)*

Many described their therapies in hospital as frightening, for example climbing stairs unaided for the first time.

##### Enduring care deficiencies

Participants that had required an ambulance at the time of their stroke all reported it had arrived quickly, and most felt that their emergency care in hospital had been good. However, three participants had been kept waiting for a long period of time in the Emergency Department (ED), and several reported waiting a long time for investigations or specialist care whilst in hospital.

*When they took her there we waited well, that took about four or five hours because the doctor, there was only one doctor on and he was rushed off his feet, he apologised, he said, they came and gave us tea we waited that long then they came back and they said he asked me what you’re asking me, start from the beginning. (Participant 16)*

Some felt the ward had been unpleasant due to very unwell patients being mixed with those who were more able bodied, noise at night, poor food and a lack of stimulation. However, one lady said this had motivated her to get better. Some had received personal care such as help with toileting from nursing staff during their stay and these individuals reported the standard of nursing care as very high.

Two participants had been admitted to a hospital far away from their homes which made it hard for their relatives to visit, one because of a bed shortage and the other because they needed specific care only available at that hospital. In the latter case the staff had made arrangements for a transfer back to the participant’s local hospital as promptly as was possible, and this had been greatly appreciated by the participant and her family.

#### Managing stroke in the community

##### Healthcare workload

Participants had seen a variety of therapists in the community following discharge, including a physiotherapist, occupational therapist, speech and language therapist, dietician, and psychologist. Therapists often worked as part of a community stroke team and some participants found it difficult to differentiate between therapists and their different roles. Most described undergoing an intense period of outpatient appointments or home visits that lasted several weeks, followed by a quieter period. Most had required therapy for limb weakness or speech difficulties.

*Oh aye, physios came. Aye they come out to the house with us, occupation therapy, they were great aye, they were great. They were coming out weekly. (Carer: three times a week.) Physio and occupational therapist, you know they done, they were a wee tag team. Aye I did, I had a busy time (Participant 10)*

Most described working hard to achieve goals by practising exercises on their own in between appointments, and making lifestyle changes such as stopping smoking and modifying their diet.

Participants described organising and collecting prescriptions. They reported varying arrangements, depending on personal circumstances. Those with poor mobility and a regular prescription tended to get this delivered weekly or monthly to their door without having to leave the house. Others relied on friends and family to pick it up for them. Most did not report difficulties, but a few struggled, for example those who were elderly.

Participants described taking numerous medications. Some mentioned drug interactions or side effects, but these had generally been dealt with by the doctor. Most said they adhered to their medication regimes, regarding these as important. Some had pill boxes that organised the tablets into daily doses to aid adherence. When discussing medication, participants often minimised the complexity of the regime until they were questioned further. Those on warfarin described having a card with information on it that they could show to health and allied health professionals such as the chiropodist or dentist, and they found this useful.

Most participants talked about managing other illnesses alongside their stroke. They described symptoms, medications, therapies, surgical procedures, and appointments. As well as chronic diseases such as diabetes, asthma and hypertension, patients talked about acute illnesses they had suffered such as gallstones or influenza, and musculoskeletal injuries they had acquired.

Participants valued their health care practitioners attempting to minimise number of appointments by dealing with multiple issues simultaneously. Participants on warfarin tended to describe more burdensome appointment schedules.

Two patients had sourced alternative therapies using their own initiative, one had researched and then started practising tai chi and the other had paid to see a practitioner who taught him the Alexander technique.

##### Enduring care deficiencies

Many participants described the period following discharge from hospital as a very difficult time. Some complained that they were discharged abruptly with a lack of follow up or support from secondary care.

*Carer: I mean they didn’t, they told us what we would need to do but they basically threw her out and that was it, that’s it, you are in charge of her….and they said oh there is this available and that available and I had to organise it all. I had to organise her physiotherapy, young person’s place over in Shettleston. (Participant 12)*

Several blamed poor communications between care providers as the reason for poor follow up, resulting in patients having to chase up appointments or results. Many with milder disabilities felt that there was a lack of community therapies available. Those with more severe disabilities tended to describe more comprehensive follow up that involved either home visits from the community stroke team or visits to the outpatient department for several weeks after discharge. Services such as cardiac rehabilitation and the day hospital were reported as helpful, and the community stroke teams were often described as excellent and well co-ordinated.

Many complained about a lack of support with regard to travelling to appointments in primary and secondary care. Patient transport systems were universally described as substandard with long waiting times and tiring, extended journeys. Appointments were occasionally missed due to patient transport delays. Those who arranged their own transport found public transport systems difficult to navigate and taxis expensive. Many patients felt they should receive more financial support from government systems. The centralisation of certain services had made travel times longer and journeys more difficult.

*I did go by the ambulance service a couple of times; you know the wee mini bus. But it was, I had to wait five hours for a lift coming back from hospital you know. And I never had any money or food or anything, you know I felt as if I was going to pass out. (Participant 10)*

Many participants felt that primary care support was lacking following discharge from hospital.

*I thought maybe a couple of days somebody just to look in. Because as you said beginning even moving, making your tea, making your dinner, I’m awful tired after that, nobody came in. (Participant 8)*

Only two participants reported good support from their family doctor immediately following discharge, which appeared to have been arranged ad hoc rather than formally requested by the hospital. Both of these participants lived in fairly affluent areas (SIMD 7). Several participants had been given a contact telephone number for secondary care to call if they needed advice or help. This appeared to make patients feel empowered and reduced waiting times for expert advice.

Regarding medications, many participants said they were happy with the regime advised by their healthcare provider. These satisfied patients were on 7–9 medications, with the exception of one who was on none. Satisfaction did not therefore appear to be related to number of tablets, although those on higher numbers did complain about having to take tablets at different times of the day, and warfarin appeared to add particular difficulties as the dose often varied from day to day and was altered frequently depending on the result of a blood test. Many reported frequent changes to medication type or dosage as problematic because this made it harder to follow a regime. Such changes had been made, for example, due to side effects or a change in clinical guidelines. A few participants also reported that changes in manufacturer had resulted in their medications changing in size, shape or colour, making adherence harder.

*Well they’ve just changed one of them, it’s the same stuff only… it’s got no days on, most of them have Monday, Tuesday, Wednesday so if I go today and I see Monday’s there I know I’ve forgotten one you know…they've changed one of them and its got no bloody days on it at all. (Participant 5)*

Participants were generally very happy with the pharmacy delivery services they were receiving; however, two did not like that they lost control of which tablets they could request each month, fearing that mistakes may be made.

Most described making adaptations to their home and many reported good assistance from health and social care; however, some reported that adaptations to the house had been difficult to organise.

*I did try and get a shower cabinet in for him…because it’s awkward for him getting in and out the bath. I’ve got the shower above the bath…but it’s very awkward for him getting in and out the bath. No we will not get it we’ve been told, there is not much, there is no money for it. (Wife, Participant 10)*

Once adaptations had been made, some participants found them unsatisfactory so had to remove them, for example one lady had been advised to replace steps outside her house with a ramp, but the ramp was not flat so she could not use her quad stick on it. Those who had to move house due to disabilities following their stroke reported long waiting times.

Several participants had home carers who visited them once or twice a day. Some helped with personal care, and others simply helped to prepare food. The amount that this was subsidised by the government varied depending on each participant’s financial situation. Those who had little support from friends and family and could not afford private help did not feel that the state funded home care was adequate for their needs.

*No because they don’t, they can’t do the things that you need. Well see like if you are, say for instance sake, windows, can’t do that. (Participant 7)*

#### Reintegrating into society

##### Healthcare workload

No participants were working at the time of interview. Two were hoping to get back to work but were awaiting assessment, and four had returned after their stroke but then subsequently retired. Depending on level of disability, some were no longer able to drive, some had been banned for a short time, and others had regained permissions though taking a driving test.

Participants described organising their finances post stroke, including sick pay from their employer or benefits from the government.

*You would send in the sick lines and they would get lost in the work and then my benefits would get stopped…. So I just had to keep on top of them and then I had phoned the DHSS.. (Participant 19)*

Enduring care deficiencies

Many reported that they had obtained walking sticks, zimmers, and wheelchairs with relative ease, however participants that required splints all described the process of obtaining these as extremely arduous and follow up poor. Some described the wheelchairs provided by the NHS (National Health Service) as difficult to use, as these required someone else to push the wheelchair from behind. Those who had tried to obtain electric ones had run into great difficulties and ended up buying these themselves. Practical advice from health professionals about coping strategies to aid mobility was appreciated by participants.

Those who had to retake their driving test gave mixed reports. Although the process was universally described as challenging, many accepted this was necessary; however, one lady complained that the wait to take a driving test was too long and the driving test centre too far away.

Participants’ experiences of applying for benefits were variable. Many had struggled to gain help, describing the process as complicated, poorly co-ordinated and difficult to understand. Some waited a long time to receive money, causing financial difficulties.

*But I had to wait months; I had to wait months to get the right money if you know what I mean, the DLA (Disability Living Allowance) and that. I had to wait months for that… (Participant 28)*

Some participants had turned to charities for help as they had received no help from health services.

#### Adjusting to life after stroke

##### Healthcare workload

Participants reported adopting coping strategies to compensate for physical disabilities and communication difficulties. Examples included planning activities ahead of time, carrying out activities more slowly, resting periodically, and communicating through friends and family.

*I don’t make it obvious that I’ve got bad balance. I tend to just touch things when I’m passing. When I go out down the steps… I put my hand on the privet hedge there, find a good strong branch that I know about as I’m going down those extra steps. (Participant 5)*

Many reported spending time gauging their physical and mental limitations and adjusting to these. They also described the difficulties of coping with slow progress. Some talked about changing their expectations of recovery as they realised their limitations.

##### Enduring care deficiencies

Some were offered psychological therapy following their stroke and most accepted this and found it useful but many were not and some felt this would have helped them.

Availability of stroke groups was often reported as poor, particularly for younger people.

*You know they were people maybe in their late seventies, eighties, some people ninety and they didn’t want me because I was only just turned sixty and they, in their eyes there was nothing wrong with me and I didn’t look to be anything wrong with me but it was all in my head, it was psychological. (Participant 20)*

Due to a lack of availability of appropriate support groups, some participants described funding their own groups.

#### Reflecting on management

##### Healthcare workload

Participants attended routine appointments with both specialists and their family practitioner or practice nurse to review their progress in the longer term. They reviewed medications and made joint healthcare decisions. They also reported using these consultations to ask about new or alternative treatments.

*I think realistically its every six months. They call me in; they call me in to do it because I’ve had a stroke for example. They call me in and say it’s time for your checks yeah, they maybe take four blood tests. They maybe take a cholesterol check, test; yeah I think it would be every six months actually.(Participant 15)*

A few expressed an interest in keeping their knowledge of stroke management up to date; however, most appeared uninterested and many would rather leave it in the hands of health professionals to alert them to new treatments.

Participants reflected on their treatments, progress and general health, some comparing their progress to others. Many felt that they were ‘lucky’ as they had not been as badly affected as others. Some monitored their ability to carry out simple tasks.

Several participants described worrying about the possibility of another stroke and spending time considering how they could modify their risk. Several reported planning how they would respond should another stroke or similar emergency occur.

*They show you how to get down on the floor and to let yourself go and to try and crawl and if you can’t crawl just lie for a minute or two, move your head to see your head is all right, move your arms try and wiggle your toes and then you bring yourself to the nearest object that is solid…That you can get to, that’s if you’ve not got this thing round your neck. (Participant 7)*

Enduring care deficiencies

Those with less severe disabilities reported a lack of short term follow up and help monitoring progress, leaving them left to gauge recovery on their own.

*No only I do keep coming back to the thought that I feel someone should have been there, someone should have been there to be able to, to be able to say to you are doing okay, just keep going the way you are. (Participant 15)*

Many patients also described longer-term follow up as poor, and this appeared to be independent of stroke severity or whether initial follow up had been poor. Poor long-term follow up resulted in medications and treatments not being reviewed for long periods of time and confidence in longer-term treatments being low.

*But I just feel as if they think well we are maintaining, I’m on a lot of medication you know and as long as nobody ever says we’ll review that or anything and I’ve been doing that I’ve been taken all that for four years, I might not need it. (Participant 20)*

A few reported deliberately not following medical advice after reflection on their own wishes. Reasons given included side effects, over complicated treatment regimens that they wished to simplify and a mismatch in ideas with health professionals.

### Patient capacity

Six main themes were identified that describe the factors that influence patient capacity to manage health problems: personal attributes and skills; physical and cognitive abilities; support network; financial status; life workload; and environment. The taxonomy of patient capacity is shown in Table 3.

**Table 3:** Taxonomy of patient capacity in stroke

Type of patient capacity	Factor affecting patient capacity
Personal attributes	• Positive characteristics e.g. resilience, independence, patience, humour and determination. • Negative characteristics e.g. disorganisation, poor engagement with health services, worry, frustration. • Knowledge and past experiences e.g. of stroke or other illnesses. • Practical capabilities e.g. physical, visual, hearing. • Cognitive capabilities e.g. memory, problem solving. • Skill set e.g. internet use.
Support network	• Friends and family that give practical and emotional support such as information gathering, medications and transport to appointments. • Volunteers / charities. • Support groups and other stroke patients. • Employment that provides a support network.
Financial status	• Financial struggles e.g. loss of income, delay in benefits. • Ability to pay for own mobility aids, adaptations, private healthcare or home care.
Life workload	• Co-morbidities. • Employment. • Dependants e.g. spouse, children.
Environment	• Geographical location e.g. distance from hospital and transport links. • Home environment e.g. stairs, access to house. • Availability of aids or gadgets.

Each theme will be described in turn with exemplar quotes.

#### Personal attributes and skills

Personality impacted on how patients managed their health and perceived their care. Those who displayed characteristics such as resilience, self-efficacy, independence, patience, and humour reported an ability to cope with treatments.

*I just kept saying I was dead positive, I just decided that you know okay I’m like this and I’ve got to do my best to get on as well as I can. (Participant 18)*

Several described using project management skills, for example to develop reminder systems for appointments. Those who had distanced themselves from health services communicated a sense of relief at avoiding treatments, yet felt more unwell and less able to cope with their everyday lives. Disorganisation, poor knowledge and negative thinking all decreased patient capacity.

*Well it's always on my mind that I could (have another stroke) because they say you take three strokes, a lot of people have told me that and I knew that myself you take three strokes. (Participant 16)*

Some lacked the necessary skills to use the internet; most participants displayed a lack of interest in this.

#### Physical and cognitive abilities

Those with severe physical disabilities had lost the ability to carry out self-care and could struggle with accessing health services.

*I won’t be stubborn I’ll say to myself I’m needing a doctor I’m going to phone so but recently I’ve been phoning the wrong numbers. I know the numbers but my hands don’t … and I phone different people in fact some people now realise that it's this silly old woman. (Participant 21)*

Those with visual, hearing or cognitive difficulties had struggled with logistical work such as organising tablets.

*Because sometimes we forget to take, she forgets to take her tablets. Now and again. Sometimes she remembers see she takes wee lapses of memory loss, she’ll maybe remember and then she’ll forget. (husband, participant 16)*

#### Support network

Participants who had close friends and family in their lives appeared to find treatment regimens less burdensome than those who coped alone. They described gaining emotional support, reassurance and help with decision making as well as practical help with housework, personal care, therapies, medications and travel to appointments.

*The warfarin one my daughter always makes sure I take it. (Participant 18)*

Many highly valued the company of other patients and visitors during their hospital stay; this had improved their mood and maintained motivation for recovery.

*So the woman from the chest, heart and stroke volunteer came every Wednesday to talk to me. And do quizzes with me and just generally ask about my family and all that sort of thing and that went on for the 13 weeks while I was still in hospital and that was great. (Participant 25)*

Women more commonly relied on their family to help with transport, whereas men more commonly obtained help with medications. Some chose to not ask friends or family for help as they worried they may be a burden; others had no-one to ask. Both of these groups described a feeling of isolation which increased treatment burden, for example a lack of help with transport meant relying on public transport or patient transport systems which were often substandard. Many described support groups as helpful. Those who were employed described their colleagues as a source of support.

*The doctor at one point sent me to a stroke society place to speak to people and it was very, very helpful because there are people there the same as you (Participant 22)*

#### Financial status

All participants were receiving treatments in the UK under the National Health Service (NHS) therefore most care was free at the point of contact. However, certain services such as home care did require payment, and some participants also chose to pay for services or treatments privately due to long NHS waiting times. Financial status varied between participants, some were able to claim sick pay through their employers but others had lost employment and suffered a reduction in income. Many were entitled to financial benefits funded by the UK government. Some found their financial difficulties a great source of stress.

*Well I’ll tell you, see the home help I pay that every month, £161.40 a month right and they were sending the bills in when I wasn’t getting money to cover it right. And I went like that I says listen nobody is getting paid, gas, electric, TV licence, nobody is getting paid...(Participant 28)*

Those with more financial resources could lessen their burden by paying for assistance in the home. Almost all of those who needed to install a walk-in shower had opted to pay themselves due to the perceived poor quality of local authority equipment and long waiting times. Some reported that they had been given financial aid by relatives to make adaptations to the house or gain mobility aids.

*I get Moira… I’ve got a little board up in the kitchen or in the hall and it tells me what days Moira is coming and how many hours…It's not through the home helps if you know what I mean… this is done privately. (Participant 21)*

#### Life workload

Some participants described areas of their life that consumed time and energy and therefore impinged on their abilities to manage their health. Comorbidities added to workload and could also result in drug-drug and disease-drug interactions.

*But then I’ve got problems with my legs, I’ve got lymphatic oedema in my legs so my legs are really heavy and I’ve got arthritis in my knees so some days it's really hard. (Participant 18)*

One lady mentioned stress at work as energy consuming and four participants reported being a carer to someone else. For those with dependents, availability of respite care increased capacity.

*My husband suffers from senile dementia so I had this to contend with and even in the hospital I’m trying to organise things that were going on you know. (Participant 22)*

#### Environment

Those who lived further away from their primary care surgery or hospital found it harder to travel to appointments, particularly if they were unable to use public transport.

*When somebody tells you we want you in (at the hospital) every morning at 10 o’clock to take your blood sample so we know what warfarin you should take tonight…and so and I thought this is ridiculous we’ve got a medical nurse, health clinic in (the local town)…why am I going in there? (Participant 24)*

Regarding the home environment, those who had been given access to mobility aids and adaptations were able to self-manage more successfully.

*She gave me a grid for my bed, for under the bed to hold onto to get up. It's a grid you put under your mattress. And I hold onto to it to get me up you know. (Participant 16)*

Technology was not commonly used; however, one man with aphasia described using an application on his tablet device to practice word recognition.

## Discussion

### Treatment burden

These findings have added to our knowledge of treatment burden in stroke. Previous systematic review showed a lack of primary studies exploring this topic [11]. Several differences were found between the treatment burdens uncovered in the systematic review [11] and patient interviews. First, analysis of interview data resulted in treatment burdens being categorised as either ‘healthcare workload’ or ‘care deficiencies’, yet in the systematic review there was no such division. Second, during the participant interviews, participants elaborated on the details of their many treatment burdens. Third, there were some new burdens found from analysis of the interviews, and these were added to the taxonomy created during the systematic review [11]. These are detailed in Additional file 4. Fourth, a few aspects of treatment burden that had been found in the review were not uncovered during participant interviews. Difficult interactions with therapists were not reported by participants; in fact, participants were more likely to describe difficult interactions with their family doctor, although this was still not common. Participants did not describe a loss of dignity on the hospital ward; instead standard of nursing care was reported as high. No differences were found between men and women regarding goal setting, information provision, or relationships with healthcare professionals. No participants were in a nursing home so treatment burdens in this setting were unable to be explored.

Differences between the two studies could have several explanations. There could be a true difference in treatment burdens between populations. This study examined a small sample from one health board area in Scotland whereas the review involved a broader exploration of papers from around the world. Additionally, due to a lack of conceptualisation of treatment burden in the literature, none of the papers in the review explored treatment burden as an explicit aim or in its entirety. This, along with the iterative nature of data collection and analysis, could explain why new treatment burdens were found in the interviews. Lastly, differences in methods of data collection could have influenced results, for example, speaking to patients directly is likely to have allowed deeper insight into the burdens experienced.

### Patient capacity

For the first time, this study explored patient capacity in stroke. Participants described six factors that affect capacity: personal attributes and skills; physical and cognitive abilities; support network; financial status; life workload; and environment. Capacity was not found to be a static entity but rather one that is ever changing depending on circumstances at any one point in time. For example, relatives who usually care for an individual may go on holiday which would diminish capacity temporarily. Additionally, similar to treatment burden, many aspects of capacity are amenable to change depending on the availability of health and social care services, for example, availability of respite care could help in the above scenario when family are away.

### Conceptual model of treatment burden and patient capacity

**Fig. 1:**
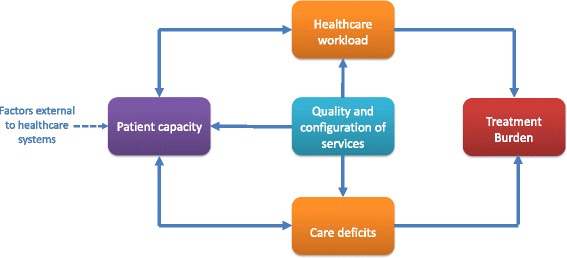
Conceptual model of treatment burden in stroke. Treatment burden arises as a consequence of healthcare workload and/or care deficiencies, which can both influence and be influenced by patient capacity. The quality and configuration of health and social care services can influence healthcare workload, care deficiencies and patient capacity (the latter is also influenced by factors external to healthcare systems such as the presence of dependents)

This work highlighted several important causal pathways. The first is that treatment burden arises because of healthcare workload and/or care deficiencies. The second is that both healthcare workload and care deficiencies can influence and be influenced by patient capacity, for example a high healthcare workload may drain time and energy, and those with more financial resources may pay for help with aspects of their care. The third is that the quality and configuration of health and social care can influence the presence of care deficiencies, the magnitude of healthcare workload and the capacity of patients to manage their health. For example, less clinical staff available on the ward may result in poor information provision from health services, which could increase workload as other sources of information are sought, and decrease capacity as those armed with less information may feel less confident to self-manage. Patient capacity can also be affected by factors independent of health services, for example those with dependents or time-demanding jobs may struggle to dedicate time to self-management. These relationships are demonstrated in the conceptual model shown in Fig. 1.

### Limitations / strengths

An important strength of this study was the limited exclusion criteria. However, as with all research studies, it is likely that the most unwell and deprived patients were ‘harder to reach’ and therefore the abler bodied and affluent over represented. The average age of participants in this study was 65, younger than the average age of those with stroke in Scotland (73 years) [22].

The investigation of capacity was limited to 14 participants from one geographical area and this would benefit from further exploration in different patient groups. Due to the limitations of qualitative work regarding generalizability, the taxonomies of treatment burden and patient capacity described here should be thought of as difficulties encountered by those with stroke that deserve further and more generalised exploration, rather than a definitive list. There was no double coding of transcripts in the second set of interviews and this could be interpreted as a limitation. Additionally, no formal respondent validation was sought after data analysis due to ethical and funding restrictions; however, feedback was informally requested from participants throughout the interviews to clarify that the true meaning of what they had said had been understood.

### How findings fit in with current knowledge

Studies on treatment burden in stroke are lacking, but there has been exploration in people with other long term conditions and multimorbidity. Sav et al. uncovered financial burden, time and travel burden, medication burden and healthcare access burden in those with multimorbidity in Australia [10]. Eton et al. created a conceptual framework of treatment burden in those with multimorbidity in the US with three distinct themes: 1) work patients must do to care for their health; 2) challenges / stressors that exacerbate burden; and 3) impacts of burden [23]. Tran et al. have created a taxonomy of treatment burden in people from 34 different countries which included a wide range of healthcare tasks, aggravators of treatment burden and patient-reported consequences of treatment burden [24]. Although these studies were not conducted in a stroke population, many findings resonate with the key themes relating to healthcare workload and care deficiencies found here.

Studies of patient capacity in stroke are scarce. An investigation of adherence to recommended treatments in stroke by Chambers et al. found that ease of medication regime, knowledge about treatments, support from health professionals and ability to adopt coping strategies all influenced adherence [25]. The Southampton Stroke Self-Management Questionnaire (SSSMQ) was recently developed to measure ability to self-manage stroke. This incorporates the patient’s beliefs, personal abilities and interactions with health professionals; however it omits the wider social influences on capacity such as financial status, social support and other personal commitments [26]. No other studies of patient capacity in stroke could be found, but investigations of people with other long term conditions have revealed that the following can influence capacity to manage healthcare workload: availability of time and knowledge; level of emotional and physical energy; the degree to which patients and practitioners agreed about the division of labour about chronic disease management; willingness to take-up types of self-management practices; financial status; and social support [9, 10, 23, 24, 27–30]. One important finding here that resonates with the work of others is that treatment burden and patient capacity are very sensitive to changes in service provision [9, 10, 23, 24, 28], important to consider when designing future health services.

### Future research

An important future step in the exploration of treatment burden and patient capacity is the creation of patient-reported measures that would enable healthcare providers to objectively assess these issues and help identify problematic areas for patients. A measure of treatment burden in people with multimorbidity was recently developed [31], however this omits stroke specific burdens such as those encountered by people with aphasia or limb weakness. It is therefore important that disease-specific measures are developed alongside generic ones to ensure that the full range of potential burdens is captured. The consequences of treatment burden in stroke merits further investigation, for example the impact on social roles, psychological health, adherence to medications, service use and the experience of family and carers. Importantly, we need to acknowledge the issue of treatment burden, and its potential negative effects on patient and caregivers. Investigating and understanding of these negative effects may give us new tools to improve patient outcomes. The taxonomies and conceptual model proposed in this paper will inform future research aimed at measuring and modifying treatment burden and patient capacity in people with stroke.

## Conclusions

This work has used qualitative methods to uncover the considerable treatment burden experienced by those with stroke, shown to be heavily influenced by the quality and configuration of health and socialcare. Taxonomies of treatment burden and patient capacity have been created along with a conceptual model of treatment burden in stroke. The future development of methods of measurement of treatment burden and patient capacity would allow these factors to be incorporated into quality measures and process indicators. It is possible that by addressing treatment burden in stroke, particularly for those who are highly comorbid, improvements can be made to the patient experience, adherence to therapies, and health-related outcomes.

## Additional files

Additional file 1: (interview schedule 1). (DOC 110 kb)

Additional file 2: (interview schedule 2). (DOC 122 kb)

Additional file 3: (participant details). (DOCX 14 kb)

Additional file 4: (new treatment burdens found from analysis of the interviews, not found in systematic review). (DOCX 16 kb)

## Abbreviations

BoTT: Burden of Treatment Theory
CuCoM: Cumulative Complexity Model
NHS: National Health Service
NPT: Normalisation Process Theory
