# Supplementary material for: A conceptual model of treatment burden and patient capacity in stroke
Source: BMC Fam Pract. 2018 Jan 9;19:9. doi: 10.1186/s12875-017-0691-4 (PMC5759246; doi:10.1186/s12875-017-0691-4)
Supplement: Supplementary file 1 — (interview schedule 1). (DOC 110 kb) [file 12875_2017_691_MOESM1_ESM.doc]

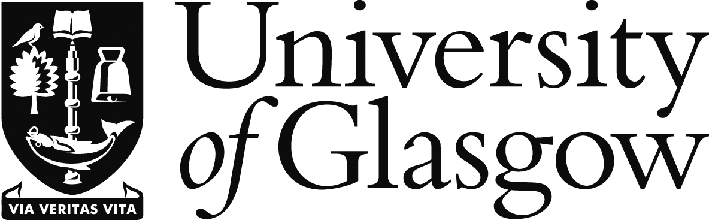

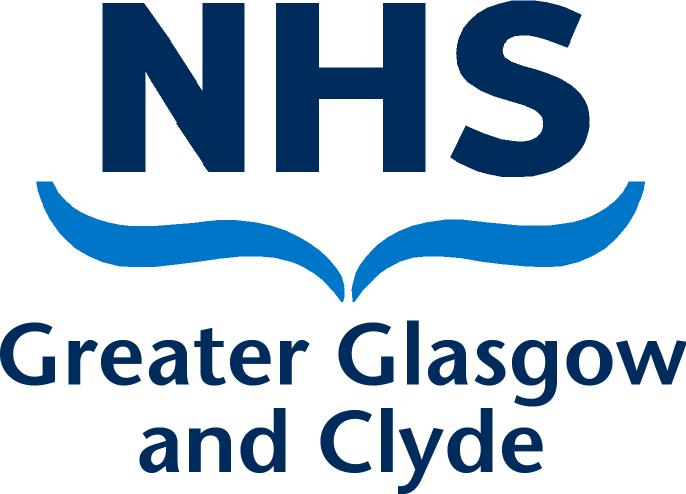


**INTERVIEW GUIDE 1: PATIENTS’ PERCEPTIONS OF TREATMENT BURDEN**

***Background Information for the Interviewer***

In situations where there is a lack of knowledge, questions will be posed in a manner which takes account of such a limitation.

The interview will be semi-structured in format, and thus the exact wording and prompts used may vary between patients to encourage the patient to share their views and allow them and opportunity to talk at length about their views about their illness and particularly their treatment and management.

***Introduction Procedure with Patients***

1. Give complete name.

2. Identify self as a researcher from the Department of General Practice and Primary Care at the University of Glasgow.

3. State that the doctors at the surgery know about the study.

4. Give short explanation of the purpose of the study:

*‘I would like you to help me understand what you feel about your stroke. I really just want to find out your views on how you have learnt to live and deal with your condition, the treatments and advice you have been given. Please feel assured that no one will be able to identify you from what you say when talking to me and everything you tell me will be treated in the strictest confidence.’*

*‘We think it is important to know not only how the illness affects your body, but also how you think the management of your condition, for example the medications you have to take and the appointments you have to attend affect your everyday life, and if so in what ways?’*

*If at any time you want to stop, or have a break, please feel free to let me know.*

*‘I will be recording the interview, so I can remember all that you have said to me.’*

**PATIENT PROFILE**

**Subject ID:**

**Age:**

**Gender:** Male ( ) Female ( )

**Length of Time Registered with Current Practice:**

Less than 5 years ( )

6 to 15 years ( )

16 to 24 years ( )

25 years + ( )

**Have you been in hospital for the condition you are taking ____________________ to treat?** Yes ( )

No ( )

**Have you seen a specialist regarding your condition?**

Yes ( )

No ( )

**Marital Status:** Single ( )

Married / Civil Partnership ( )

Living with partner ( )

Separated ( )

Divorced ( )

Widowed ( )

**Have you any Children?** Yes ( )

No ( )

**Can I ask how many?** 1 ( )

2 ( )

3 ( )

4 ( )

5+ ( )

Can you tell me whether you went on to any further education after secondary school? Yes/No

If yes, please describe.

**Date and Time of Interview:**

***COHERENCE***

*The initial questions will explore how patients make sense of stroke that is, how do they come to develop an understanding of what it means to have had a stroke and the treatment required, in terms of implications for one’s future and learning to manage their condition (coherence)?*

***The interviewer will therefore explore the following general areas in an open fashion:***

1. How the participant found out about their stroke?
2. What investigations they have had and why?
3. What treatments they have been given for what?
4. What they were first told about their treatments and by whom?
5. What they have been told since about treatments and by whom?
6. How else they have found out about their treatments? (Have they asked friends and family, read leaflets or books, watched television, looked on the internet?)
7. Whether they have received lifestyle or other advice? From whom? And what is their understanding of this advice?
8. What they have been told about risk factors and their management?
9. What they have been told to do if they have symptoms suggestive of another stroke? Or when they should seek help?

***Cognitive Participation***

The interviewer will explore how stroke patients engage with others in terms of communicating about their illness and involving others in their care (*cognitive participation*)?

***The interviewer will therefore explore the following general areas in an open fashion:***

1. When participants would ask the doctor for help, for example, make an emergency appointment? And arrangements with doctors or nurses about contacting them in an emergency?
2. How friends and family feel about their illness and its treatments?
3. Do they experience difficulty with communication due to their stroke? And what do they have to do to overcome/deal with this?
4. Whether they ask friends and family to help you with treatments and lifestyle changes or instead do everything themselves?
5. How participants organise getting prescriptions?
6. How participants organise getting to appointments?

***Collective Action***

*The interviewer will explore what activities/work do stroke patients have to undertake to help them manage their condition in practice and what challenges, if any, do their personal circumstances such as co-morbidities or social situation pose (collective action)?*

1. The interviewer will explore how difficult participants’ find it is to remember how or when to take your medicine, and what strategies, if any, participants adopt to promote medication adherence?
2. The interviewer will explore how many different types of clinics or therapists they need to attend and how they manage this? For example, how often do they go to the GP or hospital each week or month?
3. The interviewer will explore issues of access to health care? How difficult or easy it is to get appointments, access advice?
4. Participant’s confidence in their general practitioner and other health professionals will be explored and in particular their perspectives on the co-ordination of care and adequacy of communication between professionals and professionals and themselves?
5. Participants’ views on the importance, or not, of continuity of care.
6. The interviewer will explore how patients integrate their illness and its management into their social circumstances. How does it affect their life at home, leisure and social activities, or work (if relevant). For example, how, if at all, have they modified daily and social activities; or financial implications of their illness; or challenges in accessing social services support. Does it affect their diet or ability to eat or drink?
7. Have they had to get any adaptations for their home (such as a chair lift or rails), or aids to get out of the house (such as a stick)?

***Reflexive Monitoring***

*The interviewer will explore how stroke patients appraise the value of the advice provided and the therapies prescribed and does this lead them to modify their treatment regimens, if so, how and why? (reflexive monitoring)*

1. The interviewer will explore how if at all, they have adapted or modified treatments or adherence to advice and the reasons for this?
2. Participants’ thoughts/ideas about their treatments and management, including worries, will be explored. For example, whether they experience side effects or keep all their appointments and what has influenced their adherence or non adherence?
3. Whether participants discuss concerns or review their management plans with their doctor and how this has modified, if at all, how treatments will be explored? For example, are medications ever changed by the doctor or nurse and why?
4. Whether the participant’s doctor has ever made changes to their treatment regime to make it easier for them to follow?
5. Finally, what strategies, if any, participants adopt to help them keep up to date with new treatments or the latest information on their condition?

***At close of Interview***

The interviewer will ask the participant if there are any issues they would like to mention which haven’t been covered.

And thank the participant, and reiterate that all they have discussed is confidential.
