# Supplementary material for: A conceptual model of treatment burden and patient capacity in stroke
Source: BMC Fam Pract. 2018 Jan 9;19:9. doi: 10.1186/s12875-017-0691-4 (PMC5759246; doi:10.1186/s12875-017-0691-4)
Supplement: Supplementary file 2 — (interview schedule 2). (DOC 122 kb) [file 12875_2017_691_MOESM2_ESM.doc]

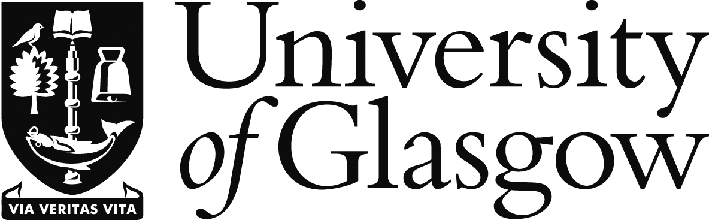

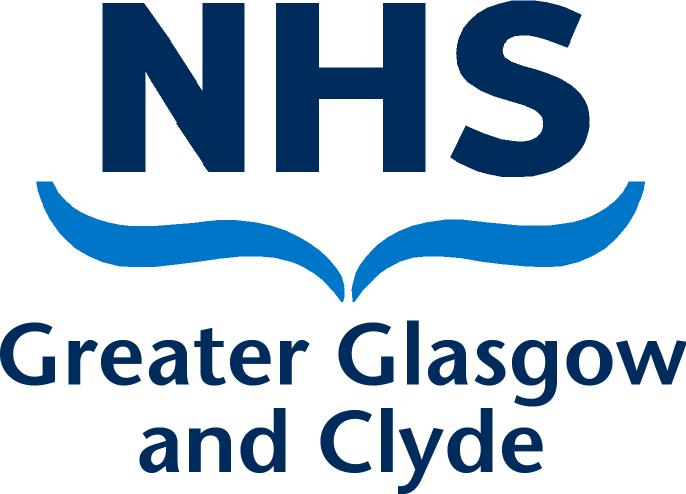


**INTERVIEW GUIDE 2: PATIENTS’ PERCEPTIONS OF TREATMENT BURDEN**

***Background Information for the Interviewer***

In situations where there is a lack of knowledge, questions will be posed in a manner which takes account of such a limitation.

The interview will be semi-structured in format, and thus the exact wording and prompts used may vary between patients to encourage the patient to share their views and allow them and opportunity to talk at length about their views about their illness and particularly their treatment and management.

***Introduction Procedure with Patients***

1. Give complete name.

2. Identify self as a researcher from the Department of General Practice and Primary Care at the University of Glasgow.

3. State that the doctors at the surgery know about the study.

4. Give short explanation of the purpose of the study:

*‘I would like you to help me understand what you feel about your stroke. I really just want to find out your views on how you have learnt to live and deal with your condition, the treatments and advice you have been given. Please feel assured that no one will be able to identify you from what you say when talking to me and everything you tell me will be treated in the strictest confidence.’*

*‘We think it is important to know not only how the illness affects your body, but also how you think the management of your condition, for example the medications you have to take and the appointments you have to attend affect your everyday life, and if so in what ways?’*

*If at any time you want to stop, or have a break, please feel free to let me know.*

*‘I will be recording the interview, so I can remember all that you have said to me.’*

**PATIENT PROFILE**

**Subject ID:**

**Age:**

**Gender:** Male ( ) Female ( )

**Length of Time Registered with Current Practice:**

Less than 5 years ( )

6 to 15 years ( )

16 to 24 years ( )

25 years + ( )

**Have you been in hospital for the condition you are taking ____________________ to treat?** Yes ( )

No ( )

**Have you seen a specialist regarding your condition?**

Yes ( )

No ( )

**Marital Status:** Single ( )

Married / Civil Partnership ( )

Living with partner ( )

Separated ( )

Divorced ( )

Widowed ( )

**Have you any Children?** Yes ( )

No ( )

**Can I ask how many?** 1 ( )

2 ( )

3 ( )

4 ( )

5+ ( )

Can you tell me whether you went on to any further education after secondary school? Yes/No

If yes, please describe.

**Date and Time of Interview:**

The interviewer will first of all explain:

We have done some previous interviews with people who have had a stroke and also looked at previous research done in this area, and would like to ask you about your thoughts on what we have found, and your own experiences.

***COHERENCE***

*Based on results from previous interviews, patients will be asked about any positive and negative experiences of learning about / making sense of their stroke, the reasons for experiences being good or bad, and any improvements that could have been made by health services.*

Interviewer will explain:

Some people who have had a stroke describe finding it difficult to gain information about their stroke, and in making sense of this information. Others feel overwhelmed by too much information. Different people prefer information being given in different forms. Some describe spending a lot of time thinking about and planning for the future, such as setting goals for their recovery.

***The interviewer will therefore explore the following general areas in an open fashion:***

# *How did you find the following experiences? Why do you think these were good or bad? How could the patient experience be improved?*

- *Reaching a diagnosis of stroke*
- *Gaining info from health professionals*

1. *How did you do the following and what could have been done to make this easier?*

- *Making sense of what a diagnosis of stroke means*
- *Understanding the roles of different health professionals involved in your care*
- *Understanding investigations and treatments that have been done / given to you*
- *Self directed research on stroke and its management (books / leaflets / internet / media/ friends and family / life experience / stroke groups)*

1. *Do you find that receiving information makes your life harder or easier? What affects this, for example, does it make a difference if the information is written or verbal?*
2. *Did you ever receive conflicting information from different sources?*
3. *Do you think about the future with regards to the following and is there any way that health services could make this easier for you:*
   - *The possibility of another stroke*
   - *How to prevent another stroke*
   - *What you would do if you thought you were having another stroke*
   - *Motivation to recover / prevent another stroke*
   - *Goal setting*
   - *The effect of stroke on your life*
   - *Coping strategies*

***Cognitive Participation***

*Based on results from previous interviews, patients will be asked about positive and negative experiences of engaging with others and organizing their stroke care, the reasons for these experiences being good or bad, and any improvements that could have been made by health services.*

Interviewer will explain:

People who have had a stroke describe seeking psychological and practical help from friends, family and other patients, and the difficulties they have with this. They also describe the difficulties they have interacting with health professionals for example speaking to doctors in the hospital, and booking appointments with their GP.

***The interviewer will therefore explore the following general areas in an open fashion:***

1. *Have your family helped with psychological and practical support such as transport, prescriptions, finances, help with therapies, aiding communication? Were there any areas of difficulty with this? Could health services help with this process in any way?*
2. *How have you found the experience of contacting your GP or the out of hours service for help or making an appointment? How could this be made easier?*
3. *How have you found the experience of contacting secondary care directly for help? How could this experience have been made easier?*
4. *Do you have difficulty using the phone?*
5. *How do you get to your appointments at your GP or at the hospital? How could this be made easier?*
6. *Did you think that health professionals spent adequate time with you in hospital? Do you have any suggestions for how this may be improved?*
7. *How do you feel you have been treated by health professionals involved in your care? Do you have confidence in these health professionals?*
8. *Is it important to you to see the same health professional i.e. is continuity important to you? What is more important: seeing a health professional quickly or seeing someone that you have seen before?*

***Collective Action***

*Based on results from previous interviews, patients will be asked about positive and negative experiences of carrying out activities involved in stroke management, the reasons for these experiences being good or bad, and any improvements that could have been made by health services.*

*.*

*Interviewer will explain:*

*People who have had a stroke sometimes describe negative experiences during their hospital stay. Some describe the time of discharge from hospital as very difficult. Once home they can find it challenging to take medications, see therapists, attend stroke groups, manage their stroke on a day to day basis. They may have to adapt their homes, move house or arrange their finances. They often find the time that they stop seeing therapists at home very difficult. Many also have other illnesses to manage at the same time.*

***The interviewer will therefore explore the following general areas in an open fashion:***

1. *What was your experience of the following in hospital and how could these experiences have been improved?*
   - *Attending hospital / emergency department at the time of having your stroke?*
   - *Being an inpatient on the ward and receiving personal care?*
   - *Undertaking therapies as an inpatient?*
   - *Discharge from hospital?*
2. *What was your experience of the following at home and how could these experiences have been improved / what would have made your life easier?*
   - *Seeing therapists either at home or in the clinic?*
   - *Practicing therapies at home on your own?*
   - *Discharge from therapists in community?*
   - *Adapting your home or moving to a suitable house?*
   - *Obtaining walking aids or braces?*
   - *Arranging social care eg carers / home helps?*
   - *Returning to driving?*
   - *Returning to work?*
   - *Finding / attending a support group?*
   - *Arranging finances / dealing with the benefits system?*
   - *Taking medications and arranging prescriptions?*
3. *Have you had to buy any of your own healthcare, social care or equipment or pay for anything else yourself?*
4. *Has there been any significant delays in your care?Whay do you think this happened?*
5. *Do you feel you received adequate psychological support during recovery?*
6. *Do you find contact with health professionals/ therapists helpful? Do you find it hard work, and if so, do you feel the work is worth it? What affects this? Overall, would you rather have seen therapists / health professionals more or less?*
7. *Do you find having several different health professionals looking after you a good or bad thing? Do you feel they communicate? How could this be improved?*
8. *Have you used technology at all during recovery e.g. an app on a smart phone? If so how did you find this?*
9. *Have you used any alternative therapies, if so, why, and how did you find the experience?*
10. *Have you made any changes to your lifestyle since your stroke and did you experience any difficulties with this? If so, what would have made your life easier?*
11. *Have you developed any coping strategies to deal with new disabilities and is there anything that would make this easier?*
12. *Do you have other illnesses to manage along with your stroke? If so, do you have any difficulties managing more than one illness? What would make your life easier?*
13. *Have you taken part in other research projects? Have you had any difficulties, and if so, what would make this easier (including this project)?*

***Reflexive Monitoring***

*Based on results from previous interviews, patients will be asked about their experiences of reflecting on their stroke management, any difficulties they have had with this, and any improvements that could have been made by health services.*

*Interviewer will explain: People who have had a stroke describe spending time reviewing their stroke care on their own and with health professionals. They make decisions about their healthcare based on their own judgments, and on advice from others.*

***The interviewer will therefore explore the following general areas in an open fashion:***

1. *How often do you see a health professional to review your stroke care? Is this too often / not often enough? Do you have any difficulties with this?*
2. *How often did you see a doctor in the hospital to review your stroke care? Was this too often / not often enough? Do you have any difficulties with this?*
3. *Do you try and keep up to date with new treatments available and if so, how, and do you have any difficulties with this?*
4. *Do you use the internet to look up information about your stroke?*
5. *Do you feel involved in decisions about your healthcare?*
6. *Are frequent changes made to your medications? How do you feel about this? What would make your life easier?*
7. *Do you monitor your own health at home in any way? What would make this easier?*
8. *How do you feel about your treatments / therapies? Have you ever stopped taking them / doing them against medical advice?*
9. *How do you feel about the progress you have made in recovering from your stroke? What could have improved this?*
10. *How have you found psychological adjustment after your stroke? Have you altered your expectations of recovery since diagnosis?*

***At close of Interview***

The interviewer will ask the participant if there are any issues they would like to mention which haven’t been covered.

And thank the participant, and reiterate that all they have discussed is confidential.
