# Supplementary material for: A conceptual model of treatment burden and patient capacity in stroke
Source: BMC Fam Pract. 2018 Jan 9;19:9. doi: 10.1186/s12875-017-0691-4 (PMC5759246; doi:10.1186/s12875-017-0691-4)
Supplement: Supplementary file 3 — (participant details). (DOCX 14 kb) [file 12875_2017_691_MOESM3_ESM.docx]

Participant details

| **Person** | **Sex** | **Age group** | **Number of co-morbidities** | **Number of medications** | **Number of strokes** | **Number of TIAs** | **Rankin score** | **SIMD decile** | **Time since diagnosis in months** |
| --- | --- | --- | --- | --- | --- | --- | --- | --- | --- |
| 1 | Male | 70-79 | 2 | 6 | 2 | 2 | 4 | 1 | 241 |
| 2 | Male | 70-79 | 2 | 4 | 1 | 0 | 2 | 8 | 241 |
| 3 | Male | 60-69 | 5 | 9 | 1 | 0 | 2 | 2 | 150 |
| 4 | Male | 70-79 | 4 | 9 | 1 | 1 | 2 | 9 | 58 |
| 5 | Male | 70-79 | 4 | 6 | 1 | 0 | 2 | 4 | 99 |
| 6 | Female | 70-79 | 2 | 8 | 1 | 0 | 0 | 2 | 145 |
| 7 | Female | 70-79 | 7 | 15 | 1 | 1 | 2 | 1 | 85 |
| 8 | Male | 70-79 | 1 | 6 | 1 | 0 | 2 | 9 | 2 |
| 9 | Male | 60-69 | 1 | 3 | 1 | 0 | 2 | 1 | 9 |
| 10 | Male | 40-49 | 2 | 4 | 1 | 0 | 2 | 2 | 217 |
| 11 | Male | 50-59 | 0 | 3 | 2 | 0 | 4 | 1 | 6 |
| 12 | Female | 60-69 | 1 | 8 | 3 | 0 | 4 | 2 | 88 |
| 13 | Male | 50-59 | 5 | 9 | 0 | 1 | 2 | 3 | 66 |
| 14 | Male | 40-49 | 0 | 3 | 1 | 0 | 3 | 1 | 7 |
| 15 | Male | 70-79 | 5 | 8 | 1 | 0 | 0 | 5 | 14 |
| 16 | Female | 70-79 | 3 | 12 | 1 | 0 | 3 | 5 | 28 |
| 17 | Female | 70-79 | 5 | 10 | 1 | 0 | 1 | 8 | 4 |
| 18 | Female | 60-69 | 3 | 7 | 1 | 0 | 3 | 8 | 64 |
| 19 | Female | 50-59 | 3 | 7 | 1 | 0 | 2 | 7 | 28 |
| 20 | Female | 60-69 | 5 | 11 | 1 | 1 | 1 | 7 | 51 |
| 21 | Female | 80-89 | 6 | 9 | 1 | 0 | 3 | 4 | 67 |
| 22 | Female | 60-69 | 0 | 0 | 1 | 0 | 0 | 7 | 25 |
| 23 | Male | 60-69 | 3 | 9 | 3 | 0 | 4 | 2 | 73 |
| 24 | Male | 80-89 | 2 | 11 | 0 | 3 | 1 | 9 | 13 |
| 25 | Female | 80-89 | 4 | 7 | 2 | 0 | 3 | 4 | 12 |
| 26 | Female | 70-79 | 1 | 11 | 1 | 0 | 2 | 9 | 131 |
| 27 | Female | 60-69 | 10 | 11 | 1 | 0 | 2 | 1 | 44 |
| 28 | Male | 50-59 | 3 | 8 | 1 | 0 | 3 | 1 | 8 |
| 29 | Male | 80-89 | 12 | 8 | 1 | 1 | 2 | 1 | 4 |
