# Supplementary material for: A conceptual model of treatment burden and patient capacity in stroke
Source: BMC Fam Pract. 2018 Jan 9;19:9. doi: 10.1186/s12875-017-0691-4 (PMC5759246; doi:10.1186/s12875-017-0691-4)
Supplement: Supplementary file 4 — (new treatment burdens found from analysis of the interviews, not found in systematic review). (DOCX 16 kb) [file 12875_2017_691_MOESM4_ESM.docx]

New aspects of treatment burden found from analysis of the patient interviews (not found in the systematic review [[11](#_ENREF_11)]).

| **Treatment burden category from Table 1** | **New treatment burden** | **Exemplar Quote** |
| --- | --- | --- |
| 2) Interacting with others | Accessing appointments in primary care | I mean I remember like if I came out and Dr X says I’ve to make an appointment for two weeks time and she would say oh right well just phone up on the morning of the appointment…You know and I would say well why would the doctor say make an appointment for two weeks time and you are telling me that I’ve to phone in the morning. (Participant 22) |
|  | Poor communication from primary care about changes to treatments | But they advised me to come off the amiodarone but that was all they said, they said they would write to my doctor. So the next thing I knew there was two items on my prescriptions. But I had, I had never heard of them you see and I thought I don’t think these are mine you know. (Participant 6) |
| 3)a) Enacting management strategies: institutional admissions. | Undergoing difficult or frightening therapies | Ah, I was, I was shaking, I was shaking I mean say it's about six stairs but I was shaking because a walking stick that was what they gave me, a walking stick and I went and then this leg was going everywhere oh I couldn’t but I done it and when you could do that you get out. (Participant 26) |
|  | Waiting as an inpatient for specialist care | And then they put me down to another ward, general and I spent a week in the general with this dizziness. And then eventually they took me for the tunnel. And they brought me out and they said you’ve had a mini stroke. (Participant 17) |
|  | Admission to a hospital far from home | And then well they took her, once they got her sort of stabilised blah blah blah because well my daughter spoke to that wee nurse, she knew her and she says it will be easier for cabbing to the Mansion House than it is from the Southern. Especially it was the winter and the snow was about that thingy. So she says we’ve got facilities, your wife is stabilised we’ve got facilities in Mansion House with speech therapists, physiotherapists and nurses and that so she went there. (Husband of Participant 16) |
| 3)b) Enacting management strategies: managing stroke in the community | Strategies to remember and organise appointments | That calendar. Every time I go to the doctors I write it down. And I leave a note in the car on my wee kind of box and I’m always in and out there putting my phone in or my fleece or whatever so I always look at it to see if there is anything in it and if I see it I say right what’s this about. (Participant 13) |
|  | Undergoing alternative therapies | I went to the Alexander Technique for a while and I think he helped me but I haven’t been for a wee while have I? He helped my balance they manipulate you, you know your shoulder and when you go in he looks at you and he says I can see you are not standing properly or you are not walking properly and he tries to get you doing things right, you know looking into a mirror and things like that and that definitely did help. (Participant 2) |
|  | Organising and collecting prescriptions | I go and pick them up myself. I phone it up or else I just drop, I was in this morning, I dropped it in and you know I ticked all the things I need and I’ll get them on Wednesday when I'm down to get my blood checked. I can’t, sometimes I’ve seen me forgetting to mark things down and getting home and saying oh jees I’ve missed that and missed this. And after a couple of days down at the doctors again. Instead of doing it all the one it’s always in bits and pieces. I do try to do everything at once and I get everything, it’s like monthly packs. (Participant 13) |
|  | Complicated medication regimes (varying instructions between tablet, frequent changes to dose, medication or manufacturer) | Well they’ve just changed one of them, it’s the same stuff only… it’s got no days on, most of them have Monday, Tuesday, Wednesday so if I go today and I see Monday’s there I know I’ve forgotten one you know…they've changed one of them and its got no bloody days on it at all. No days on it. But I checked the other ones which have the days on so I know whether I’ve taken them or not because they all get taken together. (Participant 5) |
|  | Warfarin therapy | It's under control this is quite good, well that’s eight weeks from when she first went it was maybe two weeks, three weeks because it was going up and down like a yo-yo but just recently it's went down. (Husband, Participant 16). |
|  | Travelling to appointments in primary and secondary care | I did go by the ambulance service a couple of times; you know the wee mini bus. But it was, I had to wait five hours for a lift coming back from hospital you know. And I never had any money or food or anything, you know I felt as if I was going to pass out. (Participant 10) |
|  | Poor follow up from primary care after discharge | Now I don’t know because of the illness is that all I require mainly trying to keep me more independent I don’t know but should’ve I had back up, I don’t know. I thought maybe a couple of days somebody just to look in. (Participant 8) |
| 3)d) Enacting management strategies: adjusting to life after stroke | Accessing psychological therapies | Yes I was able to go to Dr Bloomfield who is a psychotherapist. And he’s based at the Western and I went to him for 18 months and he gave me strategies to work on…Like focus on good, focus on, focus on your caravan, you feel good doing that you know on a scale of 1 to 10 how happy does that make you? These kinds of things. (Participant 20) |
| 4) Reflecting on management | Formulating an emergency plan for another stroke | They show you how to get down on the floor and to let yourself go and to try and crawl and if you can’t crawl just lie for a minute or two, move your head to see your head is all right, move your arms try and wiggle your toes and then you bring yourself to the nearest object that is solid…That you can get to, that’s if you’ve not got this thing round your neck. (Participant 7) |
|  | Enduring poor follow up to monitor progress, particularly for those who are less severely disable | But I just feel as if they think well we are maintaining, I’m on a lot of medication you know and as long as nobody ever says we’ll review that or anything and I’ve been doing that I’ve been taken all that for four years, I might not need it. (Participant 20) |
